# Supplementary material for: Collaborative intelligence in AI: Evaluating the performance of a council of AIs on the USMLE
Source: PLOS Digit Health. 2025 Oct 9;4(10):e0000787. doi: 10.1371/journal.pdig.0000787 (PMC12510544; doi:10.1371/journal.pdig.0000787)
Supplement: S2 File — (ZIP) [file pdig.0000787.s002.zip › S2 Data Raw data files containing unprocessed question inputs and LLM outputs including transcripts of deliberations/STEP3_Question-29-1_Type-MC-NJ.docx]

**Facilitator** **[Round 1]:** A 23-year-old woman is brought to the emergency department by her friend because of headache, vomiting, and diarrhea that developed rapidly 6 hours ago. She also has had associated fever, chills, generalized muscle pain, and diffuse rash. The patient previously was healthy and has had no known exposure to anyone who has been ill. Medical history is unremarkable and her only medication is an oral contraceptive. She is currently menstruating. She does not smoke cigarettes or use illicit drugs. She drinks one to two alcoholic beverages weekly. She is disoriented to person, place, and time. Vital signs are temperature 39.2°C (102.6°F), pulse 114/min, respirations 26/min, and blood pressure 82/44 mm Hg. Pulse oximetry on oxygen at 2 L/min via nasal cannula shows an oxygen saturation of 90%. Examination of the skin shows diffuse macular erythema. Lungs are clear to auscultation. Cardiac examination discloses normal heart sounds and no murmur. Bowel sounds are hypoactive. Results of laboratory studies are shown: Serum Blood ALT: 164 U/L Hemoglobin: 16.4 g/dL AST: 130 U/L WBC 14,300/mm³ with left shift Urea nitrogen: 57 mg/dL Platelet count 52,000/mm3 Creatinine 2.2 mg/dL Can you provide me a diagnosis for this case? Given your diagnosis, a response to which of the following choices below is the most likely cause of this patient's condition? (A) Bacterial endotoxin (B) Bacterial exotoxin (C) Immunoglobulin E antibody (D) Immunoglobulin G antibody (E) Viral capsid (F) Viral coat

**AI_D:** RESTATING THE QUESTION: This query involves the interpretation of clinical symptoms, physical examination findings and laboratory data of a 23-year-old woman who presents with sudden onset of headache, vomiting, diarrhea, fever, chills, muscle pain, and diffuse rash. She is disoriented and laboratory findings reveal abnormalities in enzyme levels, white blood cell count, platelet count, and kidney function. The aim is to make a diagnosis and to determine the most likely cause of the patient's condition among the options provided. PLANNING: Key issues include the onset of symptoms, the finding of a diffuse macular erythema, disorientation, and certain abnormal laboratory results. It's crucial to focus on facts related to her age, sex, the abrupt development of severe systemic symptoms, skin rash, and significant laboratory findings such as increased liver enzymes, and kidney function impairment. The pathogenesis related to the options given is also important to consider. CLINICAL REASONING: Given her fever, rash, hypotension, altered mental status, and multisystem involvement, and a history of recently starting menstruation which could mean she has been using tampons, the condition that should be considered here is Toxic Shock Syndrome (TSS). TSS is a severe, acute illness characterized by fever, rash, hypotension, and multi-organ involvement caused by toxin-producing strains of Staphylococcus aureus and less commonly, Streptococcus pyogenes. Its rapid onset and progression are related to the effects of the toxins released by these bacteria rather than the systemic spread of the bacteria themselves. CONCLUDING: The pattern develops rapidly and may cause serious health issues, even death, if not treated promptly. The accompanying laboratory findings like thrombocytopenia, increased liver enzymes, and elevated creatinine levels are concordant with this presumed diagnosis. RESPONSE: Therefore, for this case, the answer is (B) Bacterial exotoxin, as Staphylococcal and Streptococcocal TSS are primarily mediated by the exotoxins these bacteria produce, such as Toxic Shock Syndrome Toxin-1 (TSST-1).

**AI_E:** (1) RESTATING THE QUESTION: The presented case describes a 23-year-old woman who rapidly developed several symptoms, including a headache, vomiting, diarrhea, fever, chills, muscle pain, and a diffuse rash. Moreover, her disorientation and the noted changes in her vital signs and laboratory results, such as increased ALT and AST, elevated white blood cell count with left shift, increased urea nitrogen, decreased platelets, and increased creatinine suggest a systemic condition likely of infectious nature. We are asked to ascertain a possible diagnosis and identify the most probable cause, given several options: bacterial endotoxin, bacterial exotoxin, Immunoglobulin E antibody, Immunoglobulin G antibody, viral capsid, or viral coat. (2) PLANNING: The set of symptoms, particularly the diffuse macular erythema combined with the patient's high fever and the fact she is currently menstruating, cause us to consider Toxic Shock Syndrome (TSS), which is an acute, serious, and potentially fatal condition caused by toxin-producing strains of Staphylococcus aureus and Streptococcus pyogenes. (3) CLINICAL REASONING: Given the menstrual history and the rash, we should particularly consider menstrual TSS, which is often linked to tampon use during menstruation. In connection with this, bacterial exotoxins, produced by Staphylococcus aureus (such as Toxic Shock Syndrome Toxin-1, TSST-1) or Streptococcus pyogenes (pyrogenic exotoxins), are key players in the pathogenesis of TSS. These superantigens bypass the normal antigen-specific activation of T cells, leading to an overwhelming, non-specific, and systemic inflammatory response that characterizes TSS. (4) CONCLUDING: The most likely diagnosis is Menstrual Toxic Shock Syndrome, especially considering the patient's demographic, symptomatology, menstrual history, and disease course. In addition to supportive care, treatment will involve the management of any sources of infection, including tampons or nasal packing, and aggressive antibiotic therapy targeting the offending bacterial species, usually either Staphylococcus aureus or Streptococcus pyogenes. (5) RESPONSE: Drawing on the clinical reasoning above, the most probable cause of this patient's condition is (B) Bacterial exotoxin, connecting with the diagnosis of Toxic Shock Syndrome.

**AI_C:** RESTATING THE QUESTION: You want to know what diagnosis could explain the presenting symptoms and laboratory findings in a 23-year-old female patient, who is showing signs of systemic infection, disorientation, low blood pressure, increased cardiac rate and respiration rate, abnormal liver function tests, and reduced kidney function. Also, you are interested in identifying what is the most likely cause of this patient's condition from the choices provided. PLANNING: The symptoms and signs could be attributed to a systemic infection given the presence of fever, chills, generalized muscle pain, and a raised white blood cell count. Another clue for this could be the presence of a diffuse rash, which often points towards a severe infectious process. Elevated levels of blood urea nitrogen and creatinine suggest kidney involvement while raised ALT and AST levels suggest a liver problem. Considering the imminent clinical situation, we need to determine the most likely causative agent for this systemic infection from the given choices. CLINICAL REASONING: From the choices given, bacterial endotoxin and exotoxin, immunoglobulin E and G antibodies, and viral constituents (capsid and coat) could all theoretically initiate an immune response that contributes to such an illness. However, the clinical presentation here, including rapid onset, associated with diffuse macular erythema and organ dysfunction (especially kidney, manifested by elevated creatinine and BUN; and liver, manifested by raised ALT and AST) along with increased white blood cell count with left shift (suggesting a bacterial infection), is indicative of a severe condition known as Toxic Shock Syndrome (TSS). TSS, originally associated with Staphylococcus aureus and high-absorbency tampons, can be caused by other strains of bacteria as well and frequently presents with signs and symptoms as seen in our patient. The pathogenesis of TSS involves the production of bacterial toxins that act as superantigens, stimulating a massive immune response leading to cytokine storm and multiorgan failure. Staphylococcal toxic shock syndrome toxin-1, which is a known superantigen, is a bacterial exotoxin. CONCLUDING: With the clinical presentation and lab data provided, a very likely diagnosis for this patient would be Toxic Shock Syndrome. This condition can be precipitated by bacterial exotoxins, such as the Staphylococcal toxic shock syndrome toxin-1. RESPONSE: The most likely cause of this patient's condition is (B) Bacterial exotoxin, which is in line with the diagnosis of Toxic Shock Syndrome.

**AI_B:** RESTATING THE QUESTION: You want to know the potential diagnosis for a previously healthy 23-year-old female who has suddenly developed symptoms of headache, vomiting, diarrhea, fever, chills, muscle pain, rash, and disorientation. She's also showing signs of hypotension, tachycardia, and hypoxemia. Lab results indicate elevated liver enzymes (ALT, AST), increased white blood cells with a left shift, raised urea nitrogen and creatinine, and low platelet count. You're also asking which of the listed causes is most probable considering the diagnosis. PLANNING: The presented case, showing symptoms such as fever, rash, hypotension (Low Blood Pressure), and organ dysfunction (particularly kidney and liver), suggests that the woman may have 'Toxic Shock Syndrome' (TSS). It's noteworthy that TSS can be either menstrual or non-menstrual, but given that the patient is menstruating and receiving oral contraceptives, it heightens the possibility. TSS is typically triggered by certain bacterial toxins. I hypothesize that since TSS is generally related to toxins produced by bacteria, one of the bacterial toxin options (endotoxin or exotoxin) is the probable cause. CLINICAL REASONING: Toxic shock syndrome is an acute, and potentially fatal, illness caused by toxin-producing strains of Staphylococcus aureus and Streptococcus pyogenes. These bacteria produce exotoxins, which are proteins released during bacterial growth. Unlike endotoxins, which are part of the bacterial cell wall and are liberated when bacteria are lysed, exotoxins can exert their pathogenic effects at a distance from the site of bacterial growth, and they may spread through the bloodstream to distant sites, explaining the systemic symptoms observed in this patient. In the case of Staphylococcal TSS, the causative exotoxin is Toxic Shock Syndrome Toxin-1 (TSST-1). CONCLUDING: Therefore, given the patient's clinical presentation and the hallmarks of Toxic Shock Syndrome (fever, rash, hypotension and multiorgan involvement - liver and kidney in this case), the plausible diagnosis is Toxic Shock Syndrome. This diagnosis is backed further by the association between the use of tampons (particularly superabsorbent ones) and menstrual TSS instances. RESPONSE: Thus, the most fitting cause of this patient's situation suggested from the options provided is (B) Bacterial exotoxin, which is typically produced by specific strains of Staphylococcus aureus causing Toxic Shock Syndrome.

**AI_A:** 1) RESTATING THE QUESTION: A young woman with a sudden onset of systemic symptoms including headache, vomiting, diarrhea, fever, chills, muscle pain, rash, and confusion is brought to the hospital. She was previously healthy, unaware of any contact with sick individuals, and her only medication is birth control. Despite supplemental oxygen, she has a low oxygen saturation, low blood pressure, high heart rate, and elevated temperature. Her skin exhibits a widespread rash. She has increased liver enzymes, high white blood cell count with left shift, increased blood urea nitrogen (BUN) and creatinine, and a decreased platelet count. The medical query is to hypothesize the disease affecting her and determine the most probable cause among the options including bacterial endotoxin or exotoxin, immunoglobulin E or G antibodies, or viral capsid or coat. 2) PLANNING: The symptoms this woman presents are general and could reflect various pathologies, but given the speed of onset, her condition seems acute and severe. Low blood pressure, high pulse, and confusion suggest septic shock, as do the signs of organ dysfunction (increased liver enzymes, BUN and creatine and decreased platelet count). The rash may be a clue to the etiology of a possible infection, as could the recent onset of menstruation, considering her oral contraceptive use. The options provided suggest infectious causes. 3) CLINICAL REASONING: The presentation is characteristic of Toxic Shock Syndrome (TSS), which causes sudden high fever, rash and multiple organ dysfunction due to bacterial infection. TSS is commonly associated with menstruating women who use super-absorbent tampons, although the link with oral contraceptives isn't as clear. The causative organisms are usually Staphylococcus aureus or Streptococcus pyogenes, which produce an exotoxin that acts as a superantigen, triggering an overexuberant immune response. This results in systemic effects akin to septic shock. 4) CONCLUDING: The disorientation, hypoactivity of the gut, rash and fever, along with the laboratory findings of elevated liver enzymes, high WBC count with left shift, increased BUN and creatine and lowered platelets are all indicative of systemic illness, likely septic shock. Based on the clinical presentation and potential link to the menstrual cycle, the most plausible diagnosis is TSS, a condition often tied to menstruating women and linked to bacterial infection, specifically Staphylococcus aureus or Streptococcus pyogenes. 5) RESPONSE: The most probable cause of this patient's condition is a bacterial exotoxin (Choice B). Specifically, the toxins produced by Staphylococcus aureus or Streptococcus pyogenes which are typically involved in Toxic Shock Syndrome. These toxins act as superantigens, triggering an overwhelming immune response that can result in severe systemic symptoms and organ dysfunction.

**Facilitator** The consensus among the AI responses is that the 23-year-old woman's sudden onset of systemic symptoms, including headache, vomiting, diarrhea, fever, chills, muscle pain, rash, and confusion, along with her abnormal laboratory findings, suggest a severe, acute illness. The clinical presentation, including rapid onset, rash, hypotension, and multi-organ dysfunction, particularly involving the liver and kidneys, is indicative of Toxic Shock Syndrome (TSS). TSS is often associated with menstruating women, particularly those using tampons, which aligns with the patient's current menstrual status. The causative organisms are usually Staphylococcus aureus or Streptococcus pyogenes, which produce bacterial exotoxins that act as superantigens, triggering an overwhelming immune response leading to systemic symptoms and organ dysfunction. Therefore, the most probable cause of this patient's condition, given the options, is (B) Bacterial exotoxin.
